# NEIGHBORHOOD COHESION, LIVING ALONE, AND ALL-CAUSE MORTALITY IN COMMUNITY-DWELLING OLDER CHINESE AMERICANS

**DOI:** 10.1093/geroni/igac059.1094

**Published:** 2022-12-20

**Authors:** Yanping Jiang, Mengting Li, Tammy Chung

**Affiliations:** Rutgers, The State University of New Jersey, New Brunswick, New Jersey, United States; Rutgers, The State University of New Jersey, New Brunswick, New Jersey, United States; Rutgers, The State University of New Jersey, New Brunswick, New Jersey, United States

## Abstract

This study aimed to examine whether neighborhood cohesion would mitigate the adverse effect of living alone on all-cause mortality in community-dwelling older Chinese Americans. Data were drawn from the Population Study of Chinese Elderly (PINE, N = 3,157, 59-105 years, 58% female), a longitudinal study started in 2011. Mortality was tracked through December 2021 (N = 642 deceased). Cox regression indicated that neighborhood cohesion moderated the association between living alone and all-cause mortality (HR = 0.74, 95%CI [0.57, 0.97]), showing that among participants living alone (N = 678), those with high neighborhood cohesion had a 41% lower mortality risk than their counterparts with low neighorhood cohesion. In contrast, among participants living with others, those with high and low neighborhood cohesion had a similar mortality risk. These findings highlight that strong neighborhood cohesion may protect against the increased risk of premature mortality associated with living alone in older Chinese Americans.

